# Sleep paralysis in Italy: Frequency, hallucinatory experiences, and other features

**DOI:** 10.1177/1363461520909609

**Published:** 2020-03-31

**Authors:** Baland Jalal, Andrea Romanelli, Devon E. Hinton

**Affiliations:** 1 University of Cambridge; 2 University of Padua; 3 Harvard Medical School

**Keywords:** Abruzzo, cultural beliefs, hallucinations, Pandafeche, sleep paralysis

## Abstract

Previous research has found supernatural beliefs about sleep paralysis (SP) to be very prevalent in Italy, with over one third of SP sufferers believing that their SP might have been caused by a supernatural creature known locally as the *Pandafeche*. The current study further examined features of SP in Italy. All participants had experienced SP at least once in their lifetime. Participants were recruited from the general population (*N = * 67) in the region of Abruzzo. The Sleep Paralysis Experiences and Phenomenology Questionnaire (SP-EPQ) was orally administered to participants. As hypothesized, we found that Italians from the general population reported high lifetime rates of SP, prolonged duration of immobility during the event, and great fear of the experience (with as many as 42% of SP sufferers fearing that they could die from the experience), all of which were particularly elevated as compared to cultures where there are no such elaborate traditions of SP (e.g., Denmark). In addition, 78% of participants experienced some type of hallucination during their SP. The results we present here suggest that cultural beliefs about SP in Italy (e.g., as being caused by the *Pandafeche*, as reported elsewhere) potentially can profoundly shape certain aspects of the experience – a type of mind-body interaction.

## Introduction

Sleep paralysis (SP) is a state of involuntary immobility (postural atonia) occurring at sleep onset or upon awakening from sleep (Hobson, 1995; Jalal, Taylor, & Hinton, 2014; Paradis et al., 2009). Intense dreams may occur during Rapid Eye Movement (REM) sleep. To prevent our acting out of these dreams and potentially hurting ourselves, the brain temporarily paralyzes our body during REM sleep. This gross motor paralysis (i.e., atonia) entails an active inhibition of skeletal muscle tone via interneurons of the spinal cord through the neurotransmitters GABA and glycine (Brooks & Peever, 2012; Kandel, Schwartz, & Jessell, 2000). Occasionally, however, perceptual activation can take place during REM sleep, such that the person will start to awaken, yet is unable to move or speak (Paradis et al., 2009). SP may occur in the disorder of narcolepsy, a rare debilitating sleep disorder characterized by excessive daytime sleepiness (Levin, 1933). But most SP episodes occur outside narcolepsy and are not linked to serious pathology (Hufford, 1982; Sharpless & Doghramji, 2015).

Occasionally, REM mentation (that is, dreams) may intrude into emerging wakefulness. These hypnogogic (upon falling asleep) or hypnopompic (upon awakening) hallucinations may occur in all sensory modalities, and commonly include hearing footsteps and inaudible voices, and experiencing levitation and autoscopy (i.e., having out-of-body experiences). SP sufferers commonly also report sensing an invisible being or actually seeing a shadowy human-like figure descend on the body – a “bedroom intruder” that may severely terrorize the sleeper (Cheyne, Newby-Clark, & Rueffer, 1999; Cheyne, Rueffer, & Newby-Clark 1999; Cheyne & Girard, 2009; Jalal & Ramachandran, 2014; Jalal & Ramachandran, 2017; Nielsen, 2007; Solomonova et al., 2008; Simard & Nielsen, 2005). The common features of SP include the subjective experience of “sensing a terrifying presence”, hearing footsteps, and/or seeing an amorphous “intimidating” figure or shape approach the body during the event (Hufford, 1982).

These general features of SP are robust and reported by experiencers transculturally (see too, “the cultural source hypothesis”; Hufford, 1982, 1995, 2005). Thus, cultural beliefs^1^ do not dictate or drive the aspects of SP described above, which instead appear to be universal. However, different cultures explain the universal features of SP in unique ways. That is, each culture makes use of their traditional terms and of the local references available to explain these otherwise transculturally similar perceptual experiences. Supernatural explanations of SP are common, even in modern and secular countries (Hufford, 2005; Jalal, Romanelli, & Hinton, 2015), and sometimes persist in spite of learning about the neurological roots of the phenomenon. This is not surprising given the “surreal” nature of SP. Supernatural interpretations of SP include the following: in Newfoundland “Old Hag” attack (Hufford, 1982; Ness, 1978; Ness, 1985); in China, “ghost oppression” (Wing, Lee, & Chen, 1994); among the people of Japan, “*kanashibari*” (i.e., demons; Arikawa, Templer, Brown, Cannon, & Thomas-Dodson, 1999); among Cambodians, ghosts and demons (Hinton, Pich, Chhean, & Pollack, 2005, Hinton, Pich, Chhean, Pollack, & McNally, 2005); in the United States sometimes, “space alien abduction” (McNally & Clancy, 2005); in Egypt, a “*Jinn*” attack (Jalal, Simons-Rudolph, Jalal, & Hinton, 2014; on the *Jinn*, see too, Amer & Jalal, 2011); in Italy, a “*Pandafeche*” attack (Jalal, et al., 2015); in Turkey a “*Karabasan*” attack (Jalal, Eskici, Acaturk, & Hinton, in press); and among some South African indigenous groups, “*segatelelo*” (i.e., caused by black magic and dwarf-like demonic creatures called the “*tokoloshe*”; Jalal, Kruger, & Hinton, 2018).

Unsurprisingly given these features of the phenomenon, SP is greatly feared around the world (e.g., Cheyne, & Pennycook, 2013; Jalal & Hinton, 2013; Solomonova et al., 2008), and this fear often fails to diminish even after multiple episodes (Jalal & Hinton, 2013). For instance, SP has been found to evoke much greater fear than normal dream activity (Cheyne, Rueffer et al., 1999; Schredl & Doll, 1998). Some researchers have termed especially distressing SP episodes “fearful isolated sleep paralysis” (FISP) (Sharpless et al., 2010). It is believed by some that the distressing nature of the experience could perhaps in some instances – although not in the majority of cases – worsen and potentially even generate symptoms of psychopathology (once the condition becomes chronic), including anxiety and PTSD, through vicious cycles such as catastrophic cognitions leading to increased arousal, and hence to more SP, and so on (Jalal, 2016; Jalal & Hinton, 2015). However, research to date has still not clearly established whether SP can in fact generate psychopathology. Elevated rates of SP has been found in patients with PTSD (Hinton, Pich, Chhean, Pollack, 2005; Hinton, Pich, Chhean, Pollack, & McNally, 2005; Hinton, Pollack, Pich, Fama, & Barlow, 2005; Ohayon & Shapiro, 2000; Yeung, Xu, & Chang, 2005), panic disorder (Bell, Dixie-Bell, & Thompson, 1986; Bell, Hildreth, Jenkins, & Carter, 1988; Friedman & Paradis, 2002; Paradis & Friedman, 2005; Yeung et al., 2005), generalized anxiety disorder, and social anxiety (Otto et al., 2006; Simard & Nielsen, 2005). Likewise, people with elevated anxiety sensitivity (a marker of predisposition to anxiety) have high rates of SP (Ramsawh, Raffa, White, & Barlow, 2008), which may result in part from SP creating negative associations to somatic sensations. Taken together, these studies suggest that anxiety, chronic stress, and fear of anxiety symptoms may predispose to having an SP attack. These symptoms are all associated with sleep disturbances that in turn could make SP more likely. Analogously frequent idiopathic nightmares are associated with higher scores on measures of psychological disturbance (e.g., Chivers & Blagrove, 1999; Zadra & Donderi, 2000); and specifically, nightmare distress is associated with symptoms of anxiety and depression (e.g., Levin & Fireman, 2002).

Lifetime rates of SP are poorly understood, which may be one reason the phenomenon is often underdiagnosed. Notably, rates have been found to vary greatly across cultural, ethnic, and racial groups. Most studies show that 18–40% of the general population has experienced SP at least once in their lifetime. However, prevalence rates as low as 6% have been reported (Fukuda, Ogilvie, & Takeuchi, 2000; Jalal & Hinton, 2016; Wing, Chiu, Leung, & Ng, 1999; for a review, see Sharpless & Barber, 2011).

Culture may be a key factor in shaping SP episodes and, in turn, may influence its prevalence (Jalal & Hinton, 2013; Jalal & Hinton, 2016). According to the salience hypothesis, when SP is interpreted through a particular cultural lens, it may take on greater salience (Spanos, McNulty, DuBreuil, Pires, & Burgess, 1995). For example, if SP is greatly feared in a specific cultural context, catastrophic cognitions about the event (e.g., worry about a supernatural attack) could eventually lead to a type of conditioned fear (what one could call “cultural priming”). Heightened fear, through cycles of amygdaloid fear reactions and autonomic arousal, may result in more night-time awakening, and thus in more SP, and could also worsen chronic anxiety, creating a positive feedback loop (Hinton, Pich, Chhean, Pollack, 2005, Hinton, Pich, Chhean, Pollack, & McNally, 2005).

According to the panic-hallucination (PH) model of SP (Jalal, 2016), for instance, culturally driven fear might generate a panic-like reaction at the onset of the attack, resulting in the sleeper struggling to overcome the paralysis (i.e., trying to escape the attack). Such a struggle might worsen somatic symptoms, such as chest pressure, bodily tightness, and pain and spasms in limbs (see also Cheyne, Rueffer et al., 1999). Indeed, unpleasant somatic sensations coupled with fear and panic – and the distortion of “body image” (e.g., due to a functional disturbances of the temporo-parietal junction; see, Jalal & Ramachandran, 2014; Jalal & Ramachandran, 2017) – might trigger hallucinations of human-like shadowy figures, propelling a vicious fear cycle (for a related model see, Cheyne & Girard, 2009; see also Nielsen, 2007).

Consistent with the potential role of culture in shaping certain aspects of the SP event, including frequency, duration of immobility, and associated fear, traumatized Cambodians whose cultural conceptualization of the phenomenon includes dangerous visitations (e.g., from deceased relatives) and fears of multiple physical disasters (e.g., cardiac arrest) have been found to have very high rates of SP, prolonged immobility during the event, and very high rates of visual hallucinations (Hinton, Pich, Chhean, Pollack, & McNally, 2005). High rates of SP and prolonged immobility during SP experiences have also been found in Egypt, where malevolent spirit-like creatures, the *Jinn*, are thought to cause SP (Jalal, Simons-Rudolph, et al., 2014). In Egypt, SP is associated with extreme terror and fear of impending death in 50% of suffers (Jalal & Hinton, 2013). By contrast, in Denmark, where there are no elaborate supernatural beliefs about SP and where the phenomenon is regarded as an odd physiological event, lower rates and briefer SP episodes are reported, and overall fewer people (17%) fear dying from the experience.

A recent study found that over one third of SP experiencers in the Abruzzo region of Italy believed that their SP was possibly caused by the *Pandafeche*. Moreover, when asked whether they knew of a name for the experience, as many as 84% of Italian SP sufferers specifically mentioned the *Pandafeche*. This *Pandafeche* creature almost always is perceived to have ill intentions, wishing to harm the experiencer (Jalal et al., 2015). In that study, it was found that participants often believed the *Pandafeche* to be an evil witch that attacks sleepers (especially when in a supine position); to be a ghost (spirits of the deceased) or a “spirit” (unspecified supernatural creature); or to be a black cat-like creature. The link between these seemingly diverse interpretations of the *Pandafeche* is not clear. However, Abruzzo folklore provides some interesting clues about the source of these beliefs: the word *Pandafeche* is derived from the Italian word *fantasma* which means “ghost” (Giancristofaro, 2012). According to one tradition, the *Pandafeche* are witches; these witches are believed to sometimes transform themselves into cats (Procacci, 1989); and conversely, according to one folklore narration, it was believed that a woman had become a witch after eating a cat (Finamore, 1992). The *Pandafeche* has been described specifically as a black cat living in the wild Abruzzo mountains, where it has always lived (Parker, 1998). The *Pandafeche* has also been referred to in folklore as a prostitute that lies upon helpless sleepers (Politi, 1986). According to yet another tradition, the *Pandafeche* are the souls of people who died violent deaths or those of people who were wicked and evil. The *Pandafeche* is also said to have a ghost-like appearance (i.e., a vague and undefined shape), terrorizing people during dark and moonless nights^2^ (Priori, 1980). In the study by Jalal and colleagues, 18% of SP experiencers said that to prevent a *Pandafeche* attack, a broom should be placed bottom-up by the bedroom door. These data dovetail with local folklore regarding “witch visitations”; for instance, to prevent a witch from visiting one's bedroom, one should place a brush under the pillow, or behind the door or window, because a witch gets stuck counting the hairs of the brush instead of entering the room (Procacci, 1989). Similar remedies have been suggested for the *Pandafeche* in folklore tradition (see, Giancristofaro, 2012); indicating that witches perhaps in some instances may refer to the *Pandafeche* and vice versa. In brief, taken together, these findings suggest that elaborate cultural explanations of SP are common in modern day Italy, and remain an experientially salient part of Italian culture, at least as assessed in the Abruzzo region (Jalal et al., 2015; on supernatural beliefs in Italy, see also Di Nola, 1993).

In light of research pointing to elaborate cultural traditions regarding SP in Italy (i.e., in Abruzzo), it is of key importance to examine rates and features of SP in Italy, particularly in the Abruzzo region. We hypothesized that Italian SP experiencers, owing to prominent cultural traditions about SP, would have frequent SP, high rates of hallucinations, prolonged immobility, and great fear during SP, compared to cultures where there are no such elaborate traditions of the experience, e.g., Denmark (Jalal, Simons-Rudolph, et al., 2014; Jalal & Hinton, 2013).

## Methods

### Participants

Sixty-seven participants were recruited from the general population of Italy in the Abruzzo region (for details on Abruzzo, see Abruzzo Italy Quality Trade Projects, 2015). All participants had experienced SP at least once in their lifetime. Fifty-one percent of participants were male, and their ages ranged from 20–81 (*M* = 41.2, *SD* = 17.9); and years of education from 3–25 (*M* = 13.1, *SD* = 5.9). Participants were from urban (49.3%), suburban (31.3%), and rural (19.4%) geographical regions. For recruitment, see Procedures.

### Measures

#### Sleep Paralysis Experiences and Phenomenology Questionnaire

The Sleep Paralysis Experiences and Phenomenology Questionnaire (SP-EPQ) is designed by BJ and DH (the first and last author of this publication). The SP-EPQ consists of 17 items of which 12 are open-ended and five are closed-ended. The questionnaire has been used in Italy and Turkey (Jalal et al., 2015; Jalal et al., in press), and is an elaborated version of the Sleep Paralysis Questionnaire (SPQ), which has previously been used in Cambodian, Nigerian, Chinese, American, Egyptian and Danish populations (Hinton, Pich, Chhean, & Pollack, 2005; Jalal et al., 2014; Jalal, Taylor, et al., 2014; Jalal & Hinton, 2013; Ohaeri, Awadalla, Makanjuola, & Ohaeri, 2004; Yeung et al., 2005).

The first item of the SP-EPQ reads: “Some people have had the experience upon going to sleep or awakening, when they were unable to move their arms or legs or to speak, even though they wanted to do so. Have you ever had this experience?” If participants answer affirmatively to this question or are unsure, they are asked to describe their experience in more detail to confirm whether or not it was an actual event of SP (e.g., Jalal, Simons-Rudolph, et al., 2014; Jalal, Taylor et al., 2014; Jalal & Hinton, 2013; Jalal & Hinton, 2015; Jalal & Hinton, 2016; Jalal et al., in press). The SP-EPQ assesses SP frequency (lifetime, past year, and past month), triggers, time of occurrence, sleeping position and duration, associated somatic sensations and emotions, ideas of cause, hallucinatory experiences, cultural meaning of hallucinations, self-treatment and help seeking, and sources of knowledge about SP.

The SP-EPQ was translated into Italian by AR (the second author of this publication), who is a native Italian speaker (see also Jalal et al., 2015; Jalal, Simons-Rudolph, et al., 2014; Jalal & Hinton, 2013). A back translation was done to ensure that the Italian version was as close as possible to the English version.

### Procedure

Convenience sampling was used. SP sufferers were recruited through referrals from acquaintances, and through internet websites, seeking individuals who might have had “the experience upon going to sleep or awakening, when they were unable to move their arms or legs or to speak, even though they wanted to do so”. Snowballing (chain referral), a non-random convenient sampling method, was then used to increase the sample size: participants would refer other people they knew to our study (e.g., family, friends, and colleagues) who likewise might have had a similar experience in the past. This study was approved by the Institutional Review Board at the University of Padua (file number: 8542F02D6ACB07021C1AC9D3804ACB63). All participants provided either oral or written consent (the mode of consent was optional). The SP-EPQ was administered orally by the second author of the study, and the administration usually took between 20 and 25 minutes.^3^

### Data analysis

Data (i.e., vis-à-vis somatic and dissociation symptoms, and fear during SP) were analyzed using the Mann-Whitney U test, Spearman's rank correlation test and the Chi Squared test. Hypothesis-based tests were analyzed using one-tailed (directional) tests. The remaining tests were exploratory in nature; for these descriptive inquires two-tailed tests were employed.

## Results

### Rates, time of occurrence, duration and sleep position

#### Frequency of SP

The 67 SP sufferers had experienced a mean of 21.2 lifetime episodes of SP (*SD* = 43.7; range = 1-250). The distribution of lifetime episodes was as follows: one episode, 16% (11/67); 2–4 episodes, 33% (22/67); 5–20 episodes, 33% (22/67), and more than 20 episodes, 18% (12/67). Forty-three percent (29/67) of participants had experienced an SP episode during the last 12 months. The distribution of SP episodes during the past year was as follows: zero episodes, (57%); one episode, (15%), 2–4 episodes (18%), 5–10 episodes (8%), and more than 10 episodes (3%). Sixteen percent (11/67) of participants had experienced an episode during the last month. The distribution of SP episodes during the past month was as follows: zero episodes (84%), one episode (15%), and four episodes (2%).

#### Time of occurrence

SP sufferers reported their SP occurred at the following times: while falling asleep (9%), upon awakening (84%), and at both times (8%).

#### Duration of episode

SP sufferers reported the following lengths of immobility: being unable to move for less than a minute (18%), 1–5 minutes (61%), and over five minutes (21%). Self-estimated duration of episodes ranged from a few seconds to 75 minutes (*M* = 5.0, *SD* = 10.1).

#### Sleep position

Nineteen percent (13/67) of those with SP found no difference among the various sleep positions, but others did: 55% (36/67) of SP sufferers experienced SP while sleeping in a supine position, 10% (7/67) while sleeping on the stomach, 12% (8/67) while sleeping on the side, and 4% (3/67) could not remember their sleep position during SP.

### Somatic symptoms

Ninety-nine percent (66/67) of those with SP reported somatic sensations: 66% (44/67) reported palpitations; 63% (42/67) chocking sensations; 64% (42/66) trembling or shaking; 49% (33/67) sweating; 43% (29/67) chest pressure; 39% (26/67) numbness or vibrations; 27% (18/67) hot flushes and chills; 25% (17/67) shortness of breath due to the chest pressure; 19% (13/67) dizziness, lightheaded, or feeling faint; 15% (10/67) body spinning and turning; 12% (8/67) reported chest pain; and 3% (2/67) nausea and abdominal distress. On average, women reported experiencing more types of somatic sensations than males (*M* = 4.9 and *SD* = 2.4 for women vs. *M* = 3.6 and *SD* = 1.9 for men, *Z* = 2.0, *p* = .041).

Having more types of somatic sensations during SP was associated with greater fear of the experience (*ρ*_*s*_ = .5, *p* < .001, one-tailed)^4^ and greater fear of dying from SP (*ρ*_*s*_ = .4, *p* = .002, one-tailed). Having more types of somatic sensations during SP was associated with prolonged immobility during the experience (*ρ*_*s*_ = .4, *p* = .001, one-tailed). And having more types of somatic sensations during SP was associated with a higher number of lifetime episodes (*ρ*_*s*_ = .3, *p* = .017, one-tailed). Finally, having more types of somatic symptoms during SP was associated with hallucinating during the experience (*ρ*_*s*_ = .4, *p* < .001, one-tailed).

### Dissociation sensations during SP

Twenty-five percent (17/67) of participants experienced feelings of derealization (i.e., feelings of unreality) and depersonalization (i.e., detachment from oneself) during SP – we refer to these feelings collectively as “dissociation”. Six percent (4/67) of SP sufferers felt a “disconnection” between mind and body (i.e. “the mind was there, but the body was not”); one of these specifically mentioned that her body felt foreign. Three percent of participants (2/67) felt like being outside reality; one person felt like SP had “stopped time”. Three percent (2/67) said that the experience seemed unreal and inexplicable. One person had the feeling of being “different” from other people because of her SP episodes. Finally, 8% (5/67) of participants felt dissociated from themselves as a result of their out-of-the body experiences during SP; for example, one participant who watched himself from “the outside” (third-person perspective) asked himself how he could be two “persons” at once. On average, significantly more females (39.4% or 13/33) than males (11.8 % or 4/34) experienced dissociation during SP – with *χ*^2^(1) = 6.8, *p* = .009.

Individuals who experienced dissociation during SP, on average, reported marginally significantly more fear during SP, compared to those who did not report such dissociation (*M* = 3.5 and *SD* = 1.0 vs. *M* = 2.9 and *SD* = 1.3, *Z* = 1.7, *p* = .086); and they reported significantly more fear of dying (*M* = 1.9 and *SD* = 1.7 vs. *M* = 1.0 and *SD* = 1.5, *Z* = 2.5, *p* = .011]. Likewise, participants who experienced dissociation during SP reported significantly more types of somatic sensations during SP compared to those who did not report such dissociation (*M* = 5.9 and *SD* = 2.4 vs. *M* = 3.7 and *SD* = 1.9, *Z* = 3.4, *p* = .001).

### Emotions during SP

#### Fear during SP

Ninety-four percent of SP sufferers reported that they were frightened during the episodes, with the following percentages at each level of fear: 6% (4/67) a little fear, 19% (13/67) some fear, 16% (11/67) a fair amount of fear, and 52% (35/67) a lot of fear. Moreover, 42% (28/66) feared dying from SP; 8% (5/66) reported a little fear of dying, 8% (5/66) some fear, 11% (7/66) a fair amount of fear, and 17% (11/66) participants reported a lot of fear.

Fear during SP was significantly associated with lifetime episodes of SP (*ρ*_*s*_ = .2, *p* = .028, one-tailed). On average, females reported more fear during SP compared to males (*M* = 3.6 and *SD* = .7 vs. *M* = 2.5 and *SD* = 1.4, *Z* = 3.4, *p* = .001). Likewise, females reported more of fear of dying from SP compared to males (*M* = 1.8 and *SD* = 1.7 vs. *M* = 0.7 and *SD* = 1.3, *Z* = 2.8, *p* = .006).

#### Emotions other than fear

Forty-three percent (29/67) of SP sufferers reported experiencing “emotions other than fear” during their SP episodes. Ten percent (7/67) reported feeling agitation. Nine percent (6/67) reported experiencing anger and another 9% (6/67) of participants reported feeling terror or panic. Eight percent (5/67) reported anxiety; 8% (5/67) felt surprise. Six percent (4/67) felt the emotion of helplessness; 3% (2/67) experienced irritation; and 2% (1/67) reported feeling the emotion of “determination and motivation” (i.e., to overcome the hallucinated “bedroom intruder”). Two percent (1/67) reported feeling anguish; and another 2% concern. Two percent (1/67) “felt like escaping and screaming”; 2% (1/67) felt a “desire to cry”; 2% (1/67) felt “confused” about the experience; and another 2% felt “weirded out” (i.e., due to the experience being uncanny). Finally, 2% (1/67) of participants, in addition to fear, felt attraction (i.e., to the idea of becoming “bad” due to being possessed by the “devil”).

#### Positive emotions

A total of 9% (6/67) of SP sufferers reported positive emotions during their SP: 2% (1/67) reported feeling relieved when the paralysis vanished. Three percent (2/67) felt tranquility during SP (one of these because she knew SP was not dangerous; and the other because she saw the hallucination of her friend during the episode). Five percent (3/67) reported experiencing curiosity during their SP episode; and 2% (1/67) reported being “amused” during SP.

### Hallucinations during SP

Seventy-eight percent (52/67) of SP sufferers reported having hypnogogic or hypnopompic hallucinations during SP. See Table 1 for percentage of participants reporting each type of hallucination. See below for a description.

**Table 1.** Hallucinatory experiences during SP in Italy (*N* = 67).

	n	%
Sensed an unseen presence	37	55
Had visual hallucinations	25	37
“The creature” sat on their chest	6	9
Saw a shadow	17	25
Saw the shadow of a man	4	6
Saw the shadow of a woman	4	6
Saw an undefined shadow	8	12
Saw the shadows of her family members	1	2
Saw the shadow of a deformed man	1	2
Saw the shadow of a robust woman	1	2
Saw the shadow of a “faceless” woman	1	2
Saw the shadow of a big cat	1	2
Saw a person	9	13
Saw a woman dressed in old-style clothing	2	3
Saw women believed to be witches in the past	2	3
Saw his girlfriend	1	2
Saw her sister dressed in white	1	2
Saw a woman dressed in white	1	2
Saw a friend	1	2
Saw an unknown man	1	2
Saw animals and objects	4	6
Saw fluorescent, yellow-greenish eyes	1	2
Saw a tiger eating her	1	2
Saw a red hand	1	2
Saw a black brush	1	2
Saw environmental visual hallucinations	3	5
Saw “a special halo”	1	2
Saw alternating flashlights	1	2
Saw the continuation of his dream	1	2
Had auditory hallucinations	15	22
Heard environmental noises	6	9
Heard a dark noise	1	2
Heard the bulb making noises	1	2
Heard the doorbell	1	2
Heard footsteps	2	3
Heard light footsteps on the roof	1	2
Heard voices	4	6
Heard her family members calling	1	2
Heard her boyfriend calling	1	2
Heard a deep and masculine voice calling	1	2
Heard noises made by the *Pandafeche*	3	5
Heard a whistle while the *Pandafeche* was approaching	1	2
Heard a tiny noise when the *Pandafeche* jumped on her	1	2
Heard the *Pandafeche* bumping into a chair	1	2
Had tactile hallucinations	46	69
Felt “someone blocking” them	27	40
Felt choked by “the creature”	7	10
Felt to be tightly held by “the creature”	5	8
Felt the “creature's body” on them	3	5
Felt that the *Pandafeche* had immobilized their body and mind	2	3
Felt his hands blocked by the “creature”	1	2
Felt her head pressed on the pillow	1	2
Felt a “strong gravity force”	1	2
Felt turned by “the creature”	1	2
Felt the breath of the *Pandafeche* on his body	1	2
Felt the “creature” touching	14	21
Felt grasped	6	9
Felt a hand on their face suffocating them	2	3
Felt pinched by the *Pandafeche*	1	2
Felt touched on the shoulder	1	2
Felt touched on a leg	1	2
Felt embraced	1	2
Felt a caress	1	2
Felt the bed shaking	3	5
Had out-of-the-body experiences	11	16
Felt “sucked back” into his body	1	2
Saw themselves during the episode (i.e., had an experience of autoscopy)	10	15
Saw his body lying beside asking for help	1	2
Raised the head but saw it remain in its place	1	2
Saw themselves asleep	2	3
Sat on the bed but watched himself laying on the bed	1	2
Saw a tiger eating her	1	2

#### Visual hallucinations

Thirty-seven percent (25/67) had visual hallucinations during SP. Six percent (4/67) hallucinated the shadow of a male. Another 6% (4/67) saw the shadow of a woman. Twelve percent (8/67) of participants saw an undefined human shadow (i.e., a genderless shadow). Thirteen percent (9/67) hallucinated a person during their SP episodes. For instance, 3% (2/67) of participants hallucinated a women dressed in old-fashioned clothing (i.e., with a handkerchief and long skirt). One participant hallucinated fluorescent eyes (yellow-greenish) beneath their blanket; and one hallucinated being eaten by a tiger. Another person saw a red hand placed beside him, and one saw something like a black brush or hair moving in the air. One saw a particular halo beside him and another saw flashing white lights that were attributed to space aliens.

#### Auditory hallucinations

Twenty-two percent (15/67) of participants had auditory hallucinations. Nine percent (6/67) heard general noises from their environment. Three percent (2/67) heard footsteps: one participant heard “light footsteps” on the roof. Six percent (4/67) heard voices during SP: one participant heard the voices of her family members calling to her, and another heard a deep, loud, masculine voice calling her from afar.

#### Out-of-body experience hallucinations

Sixteen percent (11/67) of participants had an out-of-body experience during their SP. One participant saw his body lying beside him “asking for help but to no avail”; one raised their head during SP, but noticed the head had remained on the bed; one watched from above while a tiger was eating her body; and one felt “sucked” back into his body after leaving it temporarily, trying to escape the terror of SP.

### “Inexplicable” experiences

Six percent (4/67) reported seemingly “inexplicable” experiences related to their SP hallucinations. All these 4 participants knew each other, and all reported the same inexplicable event: they all said that, after what they called “the *Pandafeche* attack” had occurred, they found bites or bruise marks on their body.

## Discussion

Italian SP sufferers from the general population reported frequent SP (e.g., a mean total of 21.2 episodes), prolonged immobility (five minutes), and great fear of the experience; for example, 42% of SP experiencers feared dying from SP. Due to previous research pointing to elaborate cultural traditions of SP in Italy, we hypothesized to measure such a frequency of SP and features of the experience. As noted in the Introduction, a study found that over a third of Italian SP experiencers believed that their SP might have been caused by a supernatural creature known in the Abruzzo region as the *Pandafeche* (Jalal et al., 2015).

These findings suggest that culture may potentially play a pivotal role in shaping certain aspects of the SP experience in Italy. This is consistent with the salience hypothesis, which posits that when SP is interpreted through a particular cultural filter, it may become a more salient event (Spanos et al., 1995). For instance, in groups where individuals share information about SP (e.g., its cause and remedies) higher rates usually are reported. As such, the availability of cognitive categories could impact the level of attention paid to otherwise ambiguous events (Neisser, 1976). Individuals in such cultures may have been “culturally primed” to recognize and pay attention to subtle and sometimes ambiguous symptoms, such as cues of paralysis, and then seek out to confirm their culturally-laden interpretation of these by attempting to move (Spanos et al, 1995). Particularly, if the experience is greatly feared – say, as an elaborate supernatural attack – individuals might be highly motivated to escape the attack. Attempting to move (i.e., triggering the activation of motor programs) in the absence of dampening proprioceptive feedback might, in turn, generate unpleasant somatic sensations, such as pain and spasms in limbs (e.g., Cheyne, Rueffer et al., 1999; Jalal, 2016), which may feed into the hallucinatory content, and possibly prolong the immobility. Indeed, arousal mechanisms overall may lead to increased night-time awakenings, particularly during REM sleep, and in effect lead to more SP episodes (Hinton, Pich, Chhean, Pollack, & McNally, 2005). This is especially so if such distressful episodes lead to or worsen chronic anxiety, causing further sleep disturbances. “Terrorized immobility” could perhaps even serve as a trauma cue, leading to conditioned fear and consequently to amygdaloid fear activation, triggering a positive feedback loop (Bell et al., 1988; Ohayon & Shapiro, 2000; Paradis, Friedman, & Hatch, 1997; on SP as a trauma cue vis-a-vis cultural salience see, Hinton, Pich, Chhean, Pollack, & McNally, 2005). The idea of SP as a potential trauma cue is consistent with McNally and colleagues, who found that people who claim they were abducted by space aliens (but ostensibly experienced SP), show psychophysiological reactivity to audiotaped scripts describing their “alien encounters” that is either comparable with, or even exceeds, the physiological reactions of traumatized patients listening to audiotaped descriptions of their traumatic events (McNally et al., 2004). On the other hand, in Denmark for example, a culture where there are no such elaborate cultural traditions, lower lifetime rates of SP have been found (i.e., experiencers have on average six SP episodes in a lifetime), and people report shorter immobility during episodes (4.2 minutes) (Jalal & Hinton, 2013). In Denmark, only 17% of experiencers fear dying from SP, which is significantly less than among Italians (on the salience hypothesis, see also Hinton, Hufford, & Kirmayer, 2005).

To give another example where cultural priming seemingly occurs (e.g., perhaps due to expectancy-induced sensitivity to the recognition of SP and culture-driven fear), in Egypt, 41% of experiencers from the general population understand and discuss SP in the context of *Jinn* attacks and Egyptians frequently seek out traditional healing remedies to get rid of SP (Jalal, Simons-Rudolph, et al., 2014). Like Italians, Egyptians report high lifetime rates of SP (i.e., having on average 19.4 SP episodes in a lifetime) and long durations of immobility during the event (5.2 minutes) (Jalal & Hinton, 2013). Additionally, as many as 50% of Egyptian experiencers feared dying from SP. Moreover, among Egyptian SP sufferers, unlike experiencers from Denmark, believing SP to be precipitated by supernatural entities was significantly associated with fear of the experience and longer duration of immobility. Finally, Cambodians – who provide another noteworthy example – have elaborate supernatural explanations, and extremely high rates of SP and reported long durations of immobility (5.3 minutes); at a psychiatric clinic, 49% (49/100) had experienced SP in the last year, and almost all of those patients (45/49 or 92%) had experienced four or more episodes in the last year (Hinton, Pich, Chhean, Pollack, & McNally, 2005). In short, SP may take on greater salience among Italians with elaborate supernatural beliefs about the event, possibly leading to elevated rates, prolonged immobility, and great fear that could, in some instances, potentially generate symptoms of psychopathology, such as chronic anxiety.

Consistent with previous research (Fukuda et al., 1998), participants in the current study reported that their SP occurred mostly while sleeping in the supine position. This “supine position effect” has also been reported in other cultures as well. For instance, in a previous study in Italy, several participants reported that the *Pandafeche* attack could be avoided by not sleeping in the supine position (Jalal et al., 2015). The supine position might induce SP by increasing somatic sensations during sleep, due to the obstruction of airways and increased pressure on the lungs (Hiyama, Ono, Ishiwata, & Kuroda, 2000; Penzel, Möller, Becker, Knaack, & Peter, 2001; Albert & Hubermayr, 2000; Cheyne, 2002). Moreover, the majority of the participants (84%) experienced SP upon awakening, which is similar to what has been reported in other studies (e.g. Ohayon, Zulley, Guilleminault, & Smirne, 1999).

In this study, almost all the participants (99%) reported somatic sensations during SP, which included those typical of REM physiology (e.g., Douglas, 1994): feeling choked (63%), chest pressure (43%), difficulties breathing due to chest pressure (25%) and even chest pain (12%). Somatic sensations characteristic of panic reactions were also very common: sweating was reported by around half of the participants (49%) and even more participants reported palpitations (66%). In fact, studies have shown that somatic symptoms during SP often constitute a panic attack (Hinton, Pich, Chhean, Pollack, 2005; Hinton, Pich, Chhean, Pollack, & McNally, 2005). Unsurprisingly, the number of somatic sensations (i.e., more types) experienced during SP in the current study was associated with greater fear, higher lifetime rates, longer duration of immobility, and hallucinating during the event. Furthermore, interestingly, female participants reported experiencing more types of somatic sensations and also higher levels of fear during SP and were more likely to fear dying from the event. Future research should explore gender differences vis-à-vis emotional reactions to SP.

Generally, the number of people who hallucinate during SP has been found to vary in the literature. In the present study as many as 78% of participants reported some type of hallucinatory experience during SP. By contrast, one study found that 31% of Danish SP sufferers hallucinate during SP; and 34% of Egyptian SP sufferers hallucinate during their SP (Jalal & Hinton, 2013). Likewise 45% of Nigerian SP experiencers hallucinate during SP (Ohaeri et al., 2004). The highest reported rates of the hallucination is among traumatized Cambodian refugees, with 90% of those with SP reporting seeing an approaching shadow or being (Hinton, Pich, Chhean, Pollack, & McNally, 2005). Out-of-body experiences were reported by 16% of the participants and shadows were hallucinated by 25% of participants. This type of hallucination (e.g., seeing human-like shadowy figures) has been found in various cultures (e.g., Hinton, Pich, Chhean, Pollack, & McNally, 2005; Hinton, Hufford, & Kirmayer, 2005; Hufford, 1982, 2005; Jalal, Simons-Rudolph, et al., 2014), and may be a robust feature of the phenomenology of the experience, possibly due to the massive deafferention of sensory signals that occur during REM paralysis (e.g., Jalal & Ramachandran, 2014, 2017).

In the current study, some of the reported hallucinations were consistent with local cultural beliefs about the *Pandafeche*, which is believed to present itself as a witch or cat-like creature. For instance, two participants hallucinated a women dressed in old-fashioned clothing (i.e., with a handkerchief and a long skirt; that is, possibly resembling a witch) and two participants hallucinated women from the town who were thought to be witches by their respective communities in the past (Jalal et al., 2015). Moreover, one participant hallucinated fluorescent eyes (similar to those of an animal) under his blanket; another participant saw a tiger eating her,^5^ and one participant saw the shadow of a big cat (the size of an adult man) assaulting him. Taken together, these results show how pre-existing cultural frameworks and beliefs may shape the content of hallucinatory experiences during SP.

Of note, the *Pandafeche* belief can be regarded as a culture-specific nightmare interpretation in Abruzzo, Italy. Indeed, SP itself is sometimes interpreted as a dream, especially so in cultures where there is no wide-spread explanation or socio-cognitive framework for the experience (Fukuda et al., 2000). From a folkloristic perspective, the *Pandafeche* clearly refers to SP phenomenology per se; that is, a being sitting on top of a helpless sleeper (Politi, 1986), and the victim being unable to fully wake up and scream for help, for example (Giancristofaro, 2012). Nonetheless, such phenomenology may come under the general umbrella of nightmares. For instance, in Italian folk literature, the *Pandafeche* has been described as a black cat that sits on people's chest, giving them nightmares (Parker, 1998). It is thus of interest to empirically explore whether the belief in the *Pandafeche* spills over into other forms of nightmare interpretation. Accordingly, this would have implications for culture-sensitive psychiatry and treatment approaches. For example, analogously, in Islamic culture, the *Jinn* – as noted, often thought to cause SP in Egypt – are traditionally believed to influence and even cause other (non-SP) nightmares (Philips, 1989). In Egyptian culture, SP attacks represent a spiritual vulnerability that according to traditional healers requires religious intervention, such as ritualized prayer and Quranic recitation (Jalal, Simons-Rudolph, et al., 2014). On the other hand, in Italy, traditional healers do not seem to be central in the interpretation of SP attacks and nightmare-type visions. As reported in one study (Jalal et al., 2015), in Italy as opposed to in Egypt, SP experiencers do not consult traditional or religious healers about their SP experience. Indeed, most Italians have heard about SP and associated beliefs through family, friends, and other members of their community, which provides clues as to how these curious beliefs about the *Pandafeche* have persisted into modern culture. To avoid exoticism, future research should examine the degree to which the *Pandafeche* belief is steeped in local spiritual frameworks (for more details on the *Pandafeche*, see Jalal et al., 2015).

In line with previous literature (e.g., Cheyne & Pennycook, 2013; Jalal & Hinton, 2013; Solomonova et al., 2008; Sharpless et al., 2010), in the present study, the emotions experienced during SP were mostly negative. Yet surprisingly, positive emotions were reported by 9% of participants, which is noteworthy, given the underlying neurophysiology of the event and typical fear reactions driven in part by amygdaloid hyperactivation. These results suggest that SP can occasionally be a positive (or at least a non-frightening) event, supporting the idea that the condition can potentially be treated (e.g., Jalal, 2016, 2017; Sharpless & Barber, 2011; Sharpless & Grom, 2016).

We found that a small portion of the participants (6%) reported discovering physical marks on their body upon awakening from SP. These results are hard to assess objectively, given their anecdotal nature. However, body marks discovered after an SP episode might have been present before the SP attack, with the experiencer having been unaware of these; the marks might have been caused by scratching or wounding the body during a non-REM sleep phase; or the individual could show early signs of REM behavior disorder, such that he or she is able to move for short periods during REM sleep.

This study has limitations. The sample size was small and the sampling method may have resulted in a homogeneous sample, and possibly in biased results. Also it is worth stressing that even though Italy has been unified since 1861, there is diversity in terms of folklore traditions and cultural mythology. As the *Pandafeche* interpretation of SP has been reported in the Abruzzo region of Italy, it is possible that the current findings may not be generalizable to the rest of Italy. In addition, given the variance in SP rates reported in the literature, it is important that future studies clearly establish the degree to which SP rates are influenced by cultural beliefs. As we have noted previously (Jalal, Simons-Rudolph, et al., 2014), reported cultural beliefs about SP may be influenced by stigma and cultural conventions. Accordingly, future research should conduct studies using anonymous methods to investigate such cultural beliefs. It is worth noting that, while scholars across disciplines (e.g., Boyd & Richerson, 1985; Campbell, 1975; Cavalli-Sforza & Feldman, 1981; Dawkins, 1976; Hong, Morris, Chiu, & Benet-Martinez, 2000; Sperber, 1996) conceptualize culture as information that is available, accessible, and applicable to a group of individuals, culture is not uniformly spread throughout a society. That is, a given culture can be divided into subgroups or subcultures, which in turn are not homogenous, static, and closed (Fine & Kleinman, 1979). Such a conceptualization of culture as complex and dynamic should be taken into consideration when conducting research on cultural beliefs and attitudes regarding SP. That is, the complexity of culture is relevant to the idea of interpretation and meaning; indeed, ideas about SP in Italy can readily be transmitted from one subculture to another. For example, historically speaking, do cultural interpretations of SP in Italy emerge primarily from popular culture (“grassroots”) or is it at least in part transmitted down from high culture (“the elite”) to the masses? Such questions tie into arcane theoretical debates (that lie beyond the scope of this article) about the factors and processes that shape popular culture (e.g., Strinati, 2004). Finally, it is important that future research examine SP in light of natural disasters, such as earthquakes affecting the region in question; these potentially traumatic events could lead to anxiety and sleep disturbances becoming more rampant, and in turn lead to elevated SP rates.^6^

## Conclusion

In summary, as anticipated, we found that Italians from the general population reported high lifetime rates of SP, prolonged immobility, and great fear of the experience (with as many as 42% of SP sufferers fearing that they could die from the events), all at much higher rates than in cultures in which there are no such elaborate traditions of the experience (e.g., Denmark; Jalal, Simons-Rudolph, et al., 2014; Jalal & Hinton, 2013). These results suggest that culture may potentially play a key role in shaping these aspects of SP in Italy, where there are elaborate supernatural beliefs about the experience (Jalal et al., 2015). In light of these findings, future research should explore whether SP is associated with symptoms of psychopathology in Italy, such as anxiety and depression.

## Notes

1 It is worth noting that some anthropologists have pointed out that the terms “belief concepts” and “cultural meaning” are not without complexity, and that a phenomenological approach is best suited to tackle such topics (for details see, Throop, 2009; on “phenomenology,” see also Crapanzano, 2006).

2 Also, it is narrated in folklore that the *Pandafeche* does not have a preference for certain people or places (i.e., in terms of who to assault), unlike other local mythological ghost-creatures (e.g., *Farfarello* or *Mazzamurello*); that is, the *Pandafeche* will appear anywhere, whether it be in urban centers, the countryside, or “noble places,” etc. (see Priori, 1980).

3 One participant did not provide data on whether he or she experienced the somatic sensation of “trembling or shaking” during SP; and one participant did not provide data on whether he or she “feared dying from SP”.

4 When at least one of the variables was ordinal, Spearman’s rank correlation was used.

5 It should be stressed that the *Pandafeche* cultural tradition does not entail references to a “tiger” per se, but a “cat-like” creature.

6 We stress that the use of a quantitative (statistical) approach has its epistemological limitations when addressing subjective and phenomenological issues such as “cultural meaning” and “belief concepts”. As such, future studies examining cultural beliefs (e.g., the *Pandafeche* tradition vis-à-vis SP) may benefit from a qualitative or mixed methods approach (see e.g., Jalal et al., 2015).
